# Genome-Wide Comparisons of Mutations Induced by Carbon-Ion Beam and Gamma-Rays Irradiation in Rice *via* Resequencing Multiple Mutants

**DOI:** 10.3389/fpls.2019.01514

**Published:** 2019-11-28

**Authors:** Guili Yang, Wenlong Luo, Jian Zhang, Xiancheng Yan, Yan Du, Libin Zhou, Wenjian Li, Hui Wang, Zhiqiang Chen, Tao Guo

**Affiliations:** ^1^National Engineering Research Center of Plant Space Breeding, South China Agricultural University, Guangzhou, China; ^2^Institute of Modern Physics, Chinese Academy of Sciences, Lanzhou, China

**Keywords:** *Oryza sativa* L., carbon-ion beams (CIBs) irradiation, gamma rays (GRs), resequencing, single base substitution (SBS), InDels, multiple nucleotide variant (MNV), structural variant (SV)

## Abstract

Physical mutagens, such as carbon-ion beams (CIBs) and gamma rays (GRs), induce mutations with high frequency at a relatively low dose and are more user-friendly and environment-friendly in mutation breeding. Previous studies showed that CIBs induced large sized deletions and insertions, and chromosomal rearrangements, whereas GRs induce shorter deletions and insertions, and more frequent base substitutions. However, the difference on the genomic level between CIB- and GR-induced mutations remains to be clarified. In the present study, we re-sequence six mutagenized lines derived from CIB irradiation and four mutagenized lines derived from GRs. A total of 283 and 381 variations are induced in these mutants by CIBs and GRs, respectively, including single base substitutions (SBSs), small insertion and deletions (InDels), multiple nucleotide variants (MNVs). SBSs are the most abundant type of mutation and single base transition is the main form for SBSs. CIB-induced InDels accounted for 25.44% of the total variations, while GR-induced InDels accounted for 17.85%. On the contrary, the frequency of MNVs induced by GRs was approximately 2.19 times that induced by CIBs, which indicates CIBs induced increased InDels, whereas GRs induced increased MNVs. Notably, multiple base deletions (≥5 bp) were induced at a much higher frequency by CIBs than by GRs. We also find mutations induced by CIBs and GRs are unevenly distributed on chromosomes. Unusual high-frequency (HF) and low-frequency (LF) mutation regions are discovered by analyzing mutations per 1Mb along the genome. The mutation frequency within the HF regions were significantly higher than the LF regions (*P* < 0.05). A large majority of SBSs, InDels, and MNVs induced by CIBs and GRs occurred in upstream and downstream regions. Our study compares difference of mutation profiles induced by the CIB irradiation and GR on rice genomes, and give some clues for understanding the mutagenesis mechanism of physical radiation and improving the mutagenesis efficiency.

## Introduction

Creating phenotypic variation through spontaneous or artificially induced mutations and excavating mutant genes have been an important topic in plant genomic studies for decades. Under natural conditions, the mutation frequency of the genome is very low, for example, the average spontaneous mutation frequency for each regeneration is only 7 × 10^−9^ in *Arabidopsis thaliana* (Ossowski et al., 2010). However, through artificial mutagenesis treatment, the mutation frequency can be increased by tens to hundreds of times. Artificial mutations have also greatly contributed to the advancement of plant breeding (Ishikawa et al., 2012; Li et al., 2017; Kamolsukyeunyong et al., 2019).

Artificial mutations can be acquired through chemical mutagens, such as ethyl methane sulfonate (EMS), and physical mutagens, such as commonly used gamma rays (GRs) and ion beams. EMS predominantly induces point mutations throughout the genome, mainly G/C-to-A/T transitions, with high frequency (Uchida et al., 2011); however, EMS treatment of tissues or plantlets is time consuming because of its weak penetration capability (Kazama et al., 2011). The irradiation times required with physical mutagens are short; only a few seconds of irradiation are needed for imbibed seeds, tissues, and plantlets, and a few minutes are needed for dry seeds (Kazama et al., 2011).

Physical radiation includes nonionizing radiation and ionizing radiation, among which ionizing radiation carries more energy, which causes the ionization of DNA molecules and induces more lesions on DNA strands than nonionizing radiation. Direct and indirect lesions on DNA strands are likely to induce mutations, the former of which is caused by ions hitting on DNA molecules, while indirect damage is caused by free radical aggregation and cytotoxicity (Ravanat et al., 2001; Kovacs and Keresztes 2002; Alizadeh et al., 2013). The DNA damage effect of ionizing radiation is highly correlated with the linear energy transfer (LET), which refers to the average energy consumed per unit length on ionized particle tracks (Thomas et al., 2012). Ionization caused by low-LET radiation, such as GRs and X-rays, is sparsely distributed along a radiation track. However, high-LET radiation, such as protons, neutrons, and ion beams, causes dense ionization distributed along a radiation trajectory. This dense ionization leads to complex DNA damage, such as double-strand breaks (DSBs) and clustered damage (Yokoya et al., 2008). At the same absorbed dose, high-LET radiation can induce more DSBs and cluster damage than low-LET radiation. The repair of these complex DNA damages is often incomplete or even error-prone, which causes DNA mutations to be retained and inherited by offspring (Mahaney et al., 2009; Esnault et al., 2010; Eccles et al., 2011; Mladenov and Iliakis 2011).

Carbon-ion beam (CIB) irradiation, one of the typical high-LET radiation, has been widely used in mutation breeding for several plant species. CIB irradiation shows a more effective induction of DNA DSBs than other low-LET mutagenic radiations, resulting in a broad spectrum of phenotype variation (Tanaka et al., 2010; Kazama et al., 2011). Several studies have reported that CIB irradiation predominantly induces base substitutions or small insertion and deletions (InDels) (Kazama et al., 2011; Du et al., 2017). GRs are another important physical mutagen and are a typical low-LET radiation widely used in mutation breeding for almost 100 years. According to the existing theory, it can be inferred that the DNA variation induced by CIBs and GRs should be quite different. Previous studies showed that CIB irradiation induced complete deletions (whole gene sequence is deleted), whereas GRs induced partial deletions (part of gene sequence is deleted) and more frequent base substitutions (Kiefer et al., 2001; Yatagai, 2004). However, the difference at the genomic level between CIB irradiation- and GR-induced mutations remains to be clarified, such as whether CIB irradiation induces more cluster mutations, whether there is a preference for base substitutions, or whether there is an error-prone region in a chromosome. Such aspects of CIB irradiation and GRs have not been well studied; in particular, there is a lack of joint analysis of multiple mutants *via* different types of irradiation. Therefore, comparing the difference in the molecular spectrum of DNA mutations induced by CIB irradiation and GRs is important for understanding the mutagenesis mechanism of physical radiation and improving mutagenesis efficiency.

Whole-genome sequencing (WGS) is an efficient approach to characterize variants in a mutant at single-nucleotide resolution (McCallum et al., 2000; Li et al., 2017), and it provides researchers with a robust tool for analyzing the spectrum of mutations on a whole-genome scale and mapping the causal mutations more efficiently. A WGS analysis of 11 mutants in *Arabidopsis* derived from CIB irradiation indicated that single base InDels were more prevalent than larger InDels (≥2 bp) (Du et al., 2017). WGS successfully promoted the identification of induced causal mutations in plants (Tabata et al., 2013; Song et al., 2017). Therefore, utilizing WGS techniques can favorably enrich our knowledge of the actual nature of mutations induced by physical mutagens.

Rice (*Oryza sativa* L.) is a staple food for nearly half of the world's population, and it is also a model crop that has been subjected to physical mutagens (Ishikawa et al., 2012). Several studies have evaluated the mutagenic effects of CIB irradiation and GRs on rice from various aspects (Morita et al., 2009; Ishikawa et al., 2012; Techarang et al., 2018). However, the genome-wide comparisons between CIB irradiation- and GR-induced mutations in rice have not been fully characterized. In the present study, we resequenced six mutagenized lines derived from CIB irradiation and four mutagenized lines derived from GRs by WGS. The genome-wide mutation profiles induced by CIBs and GRs in rice were compared in the study to provide more information for regarding mutagenesis mechanisms. CIBs and GRs can also create various mutants, and the rice mutant collection established in our study will not only be used to facilitate genetic studies but be useful for rice breeding. Publicly access to these mutant resources will be available to the rice research community.

## Materials and Methods

### Plant Material, Mutagenesis, and Mutant Screening

Both CIB mutagenesis and GRs mutagenesis were carried out on R173, a restore line of *Oryza sativa* L. ssp. *indica*. The water content of the dry seeds was 15% which was measured by seed moisture meter. Seeds of R173 were exposed to ^12^C^6+^ ions at 80 Gy (LET = 50keV/µm) generated by the Heavy Ion Research Facility in Lanzhou at the Institute of Modern Physics, Chinese Academy of Sciences. Another set of R173 seeds were subjected to 250 Gy GRs. The irradiation doses above for both treatments were the half-lethal doses determined by our preliminary experiments. Irritated seeds were planted and the resulting M_1_ plants were grown in the rice breeding field at South China Agricultural University. M_2_ seeds from individual M_1_ plants were collected. We screened visible mutant candidates in the M_2_ or M_3_ population throughout the whole growth period and the candidate mutants were planted in separate line and bagged for selfing to get at least 500 seeds in the subsequent M_3_ to M_6_ generation. Finally, six lines (including five M_4_ and 1 M_5_) induced by CIB irradiation and four M_6_ lines induced by GRs were used for sequencing.

### Illumina Paired-End Sequencing and Genomic Variant Detection

Genomic DNA was extracted from a single plant of each mutant line. The rice genomic DNA was extracted using the cetyl-trimethylammonium bromide method and quantified using a NanoDrop ND-1000 spectrophotometer (Thermo Scientific, Wilmington, USA). The DNA was then subjected to fragmentation, library construction and paired-end sequencing using an Illumina HiSeq 2500 sequencer according to the manufacturer's instructions. The insert sizes were 300 to 500 bp, and read length was 100 bp.

Sequencing data of the mutant and wild-type (WT) individuals generated by the Illumina HiSeq platform were mapped to the Nipponbare reference genome (IRSGP-1.0) by Burrows-Wheeler Alignment tool (Li and Durbin 2009) following default parameters, and then sorted and indexed by SAMtools (Li et al., 2009) to produce binary alignment files. Three different software programs, VarScan 2 (Koboldt et al., 2012), Platypus (Rimmer et al., 2014) and DELLY (Rausch et al., 2012), were used to identify the mutations from these two datasets. Among these software, Varscan 2 detected single base substitutions (SBSs) and small InDels (insertions or deletions <10 bp), Platypus detected multiple nucleotide variants (MNVs), and Delly detected large InDels (insertions or deletions >50 bp). VarScan2 and Delly detected the mutations using default parameters, and Platypus was used in assembly mode following the parameters of (– assemble 1, – assemble BrokenPairs = 1, – assemble All = 1). According to previous studies, an insertion/deletion size of 1 to 10 bp is defined as an InDel, and an insertion/deletion size of more than 50 bp is defined as an SV (Alkan et al., 2011). MNV means substitution mutations occur at consecutive bases, these tandem substitutions of MNV can be produced within several helical turns of the DNA by ionizing radiation (Georgakilas et al., 2013; Wei et al., 2015; Besenbacher et al., 2016). To provide an accurate description, the mutation types analyzed in this study were grouped into four categories: SBS, MNV, InDel, and SV.

Based on the four types of detected mutations, SnpSift (Cingolani et al., 2012a) was used to filter the reliable mutations of each mutagenesis progeny: for one dataset, only those mutation sites with a variant allele frequency by read count between 25% and 100% in one sample, 0% to 10% in other samples were reserved, and variants with a variant allele frequency of ≥75% were considered homozygous. The sites shared by the mutant lines were called background mutations. After filtering the reliable mutation sites, Integrative genomics viewer was used to verify the mutation sites by visual analysis (Robinson et al., 2011). All data were computed by an IBM X3850 server and the mutation distribution figure was drawn using Circos (version 0.66) (Krzywinski et al., 2009).

### Variant Annotation and Effect Prediction

To identify genes affected by each induced mutation, we used the SnpEff (Cingolani et al., 2012b) and rice7 databases (https://sourceforge.net/projects/snpeff/files/databases/v4_2/).

### Verification of the Mutation Sites by Mutation Mapping

To verify the reliability of our resequencing data, we confirm one of the mutations through combination of bulked segregant analysis (BSA) and resequencing data. A GR-induced mutant H404 was crossed with a *japonica* rice variety 02428 with normal floret and an F_2_ (H404/02428) was successfully constructed. Four hundred sixty-five SSR markers were used for analysis. SSR markers which are polymorphic between H403 and 02428 will be used for the following BSA. The segregation ratio of the phenotype of normal floret or abnormal floret was investigated by χ^2^ test. Two phenotypic extreme pools were generated by mix equal amount of leaf from 20 plants with abnormal floret (R-pool) and 20 plants with normal floret (D-pool), respectively. The above selected polymorphic SSRs were used to analyze the two pools. The markers associated with the phenotype of interest were screened out, and we check the physical position of the associated markers on the GRAMENE (http://archive.gramene.org/). Finally the nonsense mutation sites around the associated markers were investigated in the resequencing data.

### Data Availability and Statistics

The WGS data reported in this study have been deposited in the Genome Sequence Archive (Genomics, Proteomics & Bioinformatics, 2017) in the BIG Data Center, Beijing Institute of Genomics (BIG), Chinese Academy of Sciences, under accession number CRA000464 and are publicly accessible at http://bigd.big.ac.cn/gsa. Independent-samples *t* test was used for statistics in our study.

## Results

### Summarized Information of CIB- and GR-Induced Mutations

**Figure 1:**
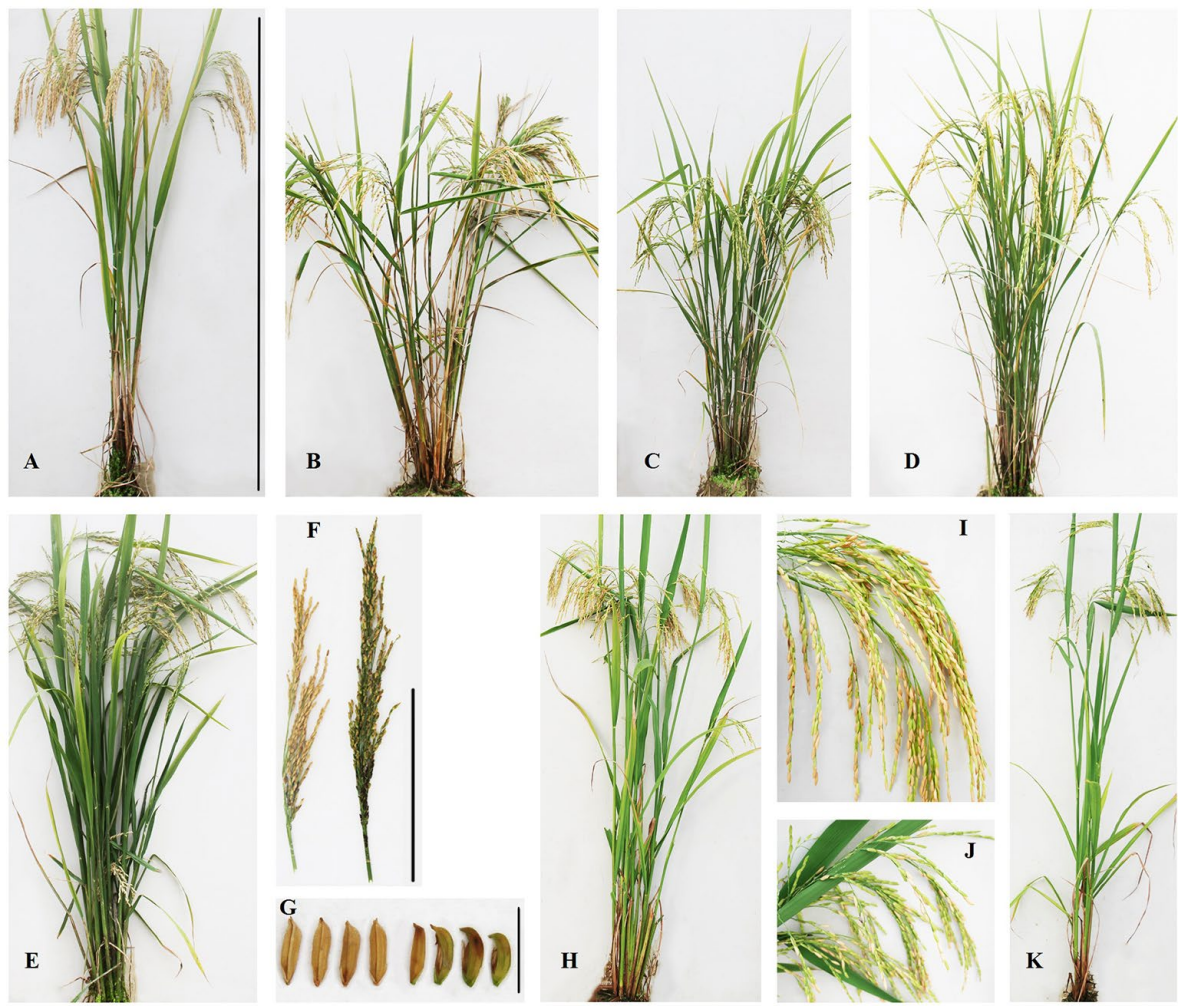
Phenotypes of the mutant lines induced by CIBs and GRs. **(A)**: R173, Wild type (Bar: 100cm); **(B)**: H409 (semi-dwarf); **(C)**: H410 (semi-dwarf); **(D)**: H411 (semi-dwarf); **(E)**: H404 (abnormal floret); **(F)**: The panicles of H404 (right) and WT (left) (Bar: 20cm); **(G)**: The spikelet of H404 (right) and WT (left) (Bar: 10cm); **(H)**: H634 (lower seed set); **(I)**: The panicle of H634; **(J)**: The panicle of H635; **(K)**: H635 (lower seed set).

CIB- and GR-treated 1,000 seeds (M_0_) each were planted, and we acquired 465 and 532 M_1_ plants, respectively. Then M_2_ seeds from individual M_1_ plants were collected. Phenotypic screening for visible mutant candidates in the M_2_ or M_3_ population was throughout the whole growth period, and six lines (H633, H634, H635, H636, H639, H640) induced by CIB irradiation and four M_6_ lines (H404, H409, H410, and H411) induced by GRs were used for sequencing in the end. All the mutants displayed visible and heritable traits, and the phenotypes of the lines are shown in **Figure 1**. All the six CIB-induced lines displayed lower seed sets. The four GR-induced mutants showed semi-dwarf, or abnormal floret.

To investigate the different effects of CIB and GR irradiation on the rice genome, we sequenced the screened mutant lines and the nonirradiated parental line. Our subsequent analyses were based on the six mutant lines induced by CIBs, four mutant lines induced by GRs and the WT were chosen for whole-genome resequencing. The summarized resequencing information of all the samples is listed in **Table 1**. On average, 9.37 Gb were obtained for each line and mapped onto the Nipponbare reference genome, resulting in an average sequencing depth of 25.11-fold.

**Table 1:** The phenotypes and resequencing information of the mutant lines.

Treatment	Line	Phenotype description	Sequenced generation	Clean data (G)	Theoretical sequencing depth
CK	R173	Wild type	**/**	45	121
CIB	H633	Lower seed set	M4	11	31
	H634	Lower seed set	M4	8	23
	H635	Lower seed set	M5	10	26
	H636	Lower seed set	M5	12	31
	H639	Lower seed set	M4	11	30
	H640	Lower seed set	M4	12	31
GR	H404	Abnormal floret	M6	8	21
	H409	Semi-dwarf	M6	7	20
	H410	Semi-dwarf	M6	7	19
	H411	Semi-dwarf	M6	7	19

CK represents control.

Based on the classification of the mutation types as described above, the reliable mutation data of 10 mutants were sorted out as shown in **Table 2**. A total of 283 and 381 variations were detected in these mutant lines by CIBs and GRs, respectively. We identified 175 SBSs, 72 InDels, and 36 MNVs in the CIB-induced mutants and 259 SBSs, 68 InDels, and 54 MNVs in the GR-induced mutants. There were no detected SVs either in GR or CIB treatment. The number of mutations in most mutants was less than 100, and only two GR-induced mutants, H404 and H409, had more than 100 mutations. The SBSs in most of the mutants accounted for more than 60% of the total mutations, indicating that SBSs were the dominant type of mutation.

**Table 2:** Summary of mutation information of individual lines.

Treatment	Line	Total	Number of mutation				Proportion of each mutation (%)			
			SBSs	MNVs	InDels	SVs	SBSs	MNVs	InDels	SVs
CIB	H633	52	36	6	10	0	69.23	11.54	19.23	0.00
	H634	46	27	5	14	0	58.70	10.87	30.43	0.00
	H635	51	29	13	9	0	56.86	25.49	17.65	0.00
	H636	71	38	11	22	0	53.52	15.49	30.99	0.00
	H639	35	24	1	10	0	68.57	2.86	28.57	0.00
	H640	28	21	1	6	0	75.00	3.57	21.43	0.00
	average	47.17	29.17	6.17	11.83	0.00	63.65	11.64	24.72	0.00
GR	H404	109	64	17	28	0	58.72	15.60	25.69	0.00
	H409	109	76	14	19	0	69.72	12.84	17.43	0.00
	H410	94	63	11	20	0	67.02	11.70	21.28	0.00
	H411	69	57	12	0	0	82.61	17.39	0.00	0.00
	average	95.25	65.00	13.50	16.75	0.00	69.52	14.38	16.10	0.00

SBSs, single base substitutions; MNVs, multiple nucleotide variants; Indels, insertion/deletion size of 1 to 50 bp; SVs, structural variant, insertion/deletion size >50 bp.

**Figure 2:**
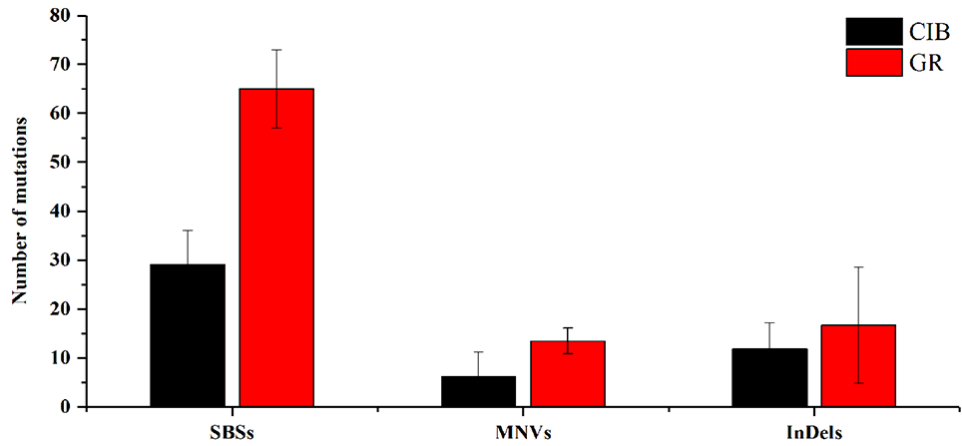
Average numbers of different mutation types induced by CIBs and GRs in all the mutants. SBSs, single base substitutions; MNVs, multiple nucleotide variants. **Represents significant at 0.01 level; *represents significant at 0.05 level.

For a single mutant, the mutations detected in GR-induced mutants were more than that detected in CIB-induced mutants (**Figure 2**). For example, on average, 65.00 SBSs, 13.50 MNVs, and 16.75 InDels were detected, in each GR-induced mutant, while 29.17 SBSs, 6.17 MNVs, and 11.83 InDels were detected in each CIB-induced mutant. Although more InDels were detected in each GR-mutant, the proportion of InDels detected in CIB-induced mutants was higher than in GR-induced mutants. CIB-induced InDels accounted for 25.44% of the total variation, while GR-induced InDels accounted for 17.85%. Moreover, we analyzed the number of MNVs. And we found that the frequency of MNVs detected in the GR-induced mutants was approximately 2.19 times that in the CIB-induced mutants. For a single mutant, the average number of MNVs in the GR-induced mutants was 13.50, while that of MNVs in the CIB-induced mutants was 6.17.

The above results showed that SBSs are prevalent in both CIB- and GR-induced mutants. Comparatively, CIB irradiation may be inclined to induce more InDels, whereas GRs may be prone to induce more MNVs.

### Frequency and Distribution of Mutations for Different Mutagenesis Treatments

#### Distribution of Mutations

**Figure 3:**
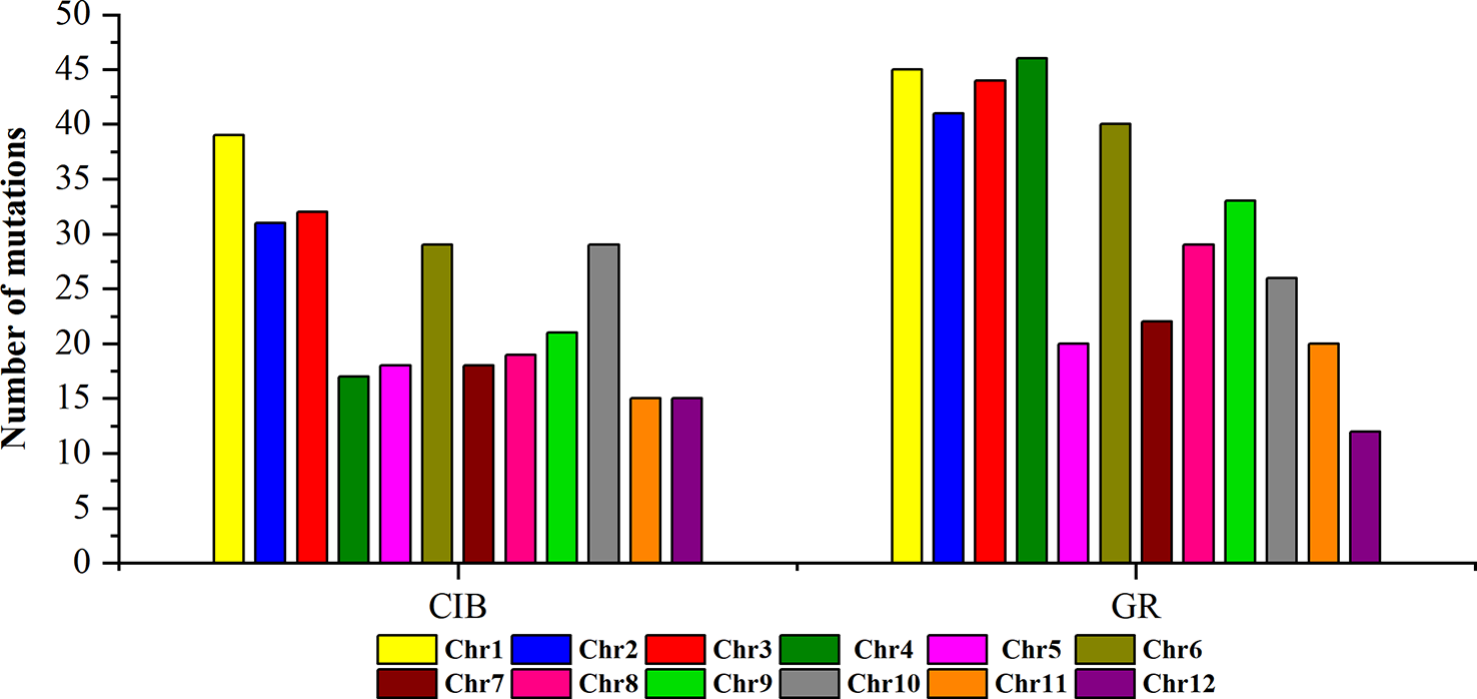
The numbers of mutations detected for on different chromosomes induced by CIBs and GRs in all the mutants.

**Figure 4:**
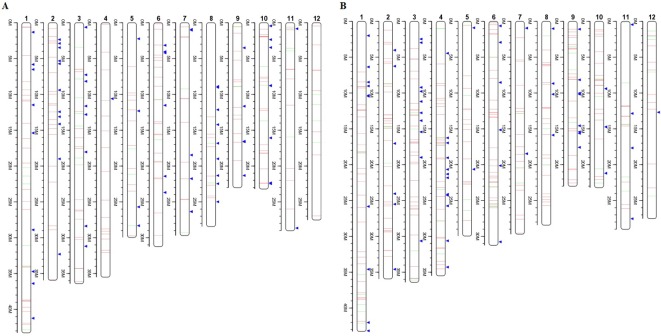
Distributions of different mutation types on the each chromosome. **(A)**: Distribution of mutations from all the six CIB CIB-induced lines; **(B)**: Distribution of mutations from all the four GR-induced lines. Red lines represent SBSs, green lines represent MNVs, and blue triangles represent Indels.

To investigate the different effects of CIB and GR irradiation on the rice genome, we mix the mutations detected in every single mutant induced by two mutagens. On the whole, the number of mutations on different chromosomes was nearly proportional to their length (**Figure 3**). However, mutation frequency varied among the chromosomes (**Table S1**). For the GR treatment, mutations occurred on average about every 697.36 kb on Chr. 9 but about every 2,294.32 kb on Chr. 12. For the CIB treatment, the highest mutation frequency, one per 800.25 kb, occurred on Chr. 10, and the lowest mutation frequency, one per 2,088.40 kb, occurred on Chr. 4. Moreover, we compared the physical locations of all of the different mutations. We found overlap between SBSs and MNVs was rare, but the adjacent areas of InDels were more likely accompanied by SBSs (**Figure 4**). In GR and CIB irradiation, the proportions of InDels accompanied by SBSs were 23.8% and 23.9%, respectively.

**Figure 5:**
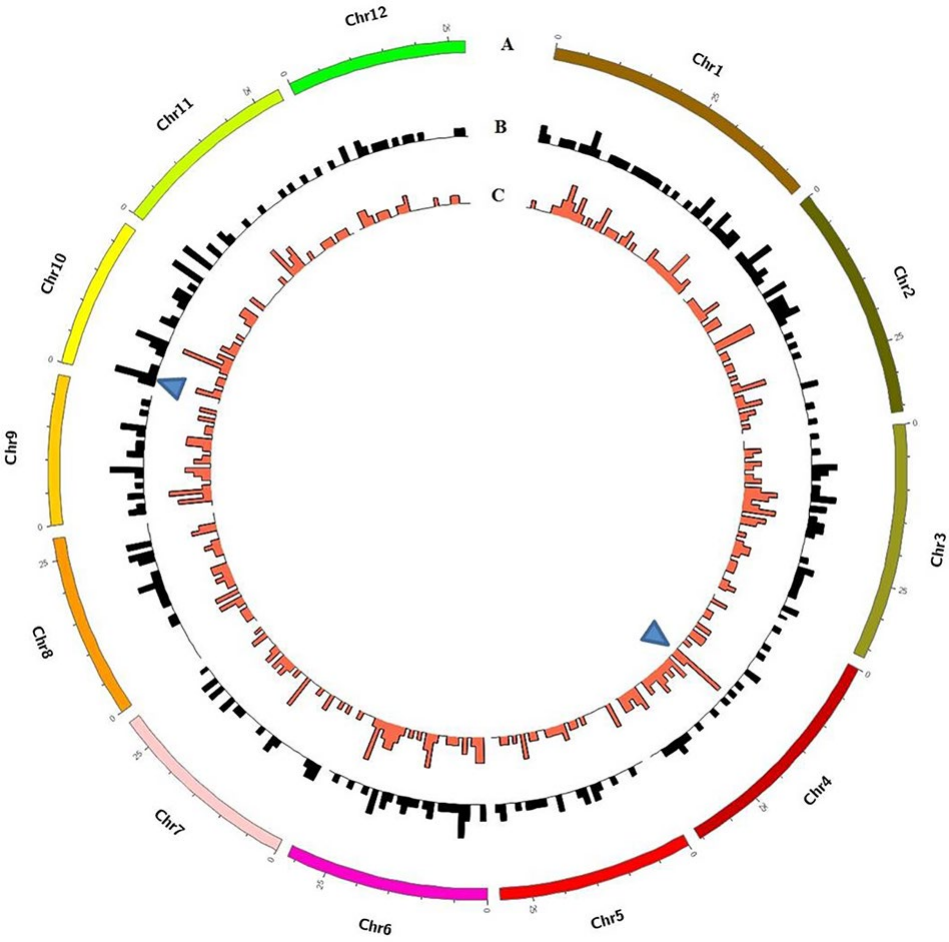
Genome-wide Characterization of Mutations in rice induced by and CIBs and GRs. **(A)** Representation of the 12 rice chromosomes on an Mb scale. **(B)** CIB-induced mutations in non-overlapping 100-kb windows. **(C)** GR-induced mutations in non-overlapping 100-kb windows. Blue triangles represent clustered mutation regions.

**Figure 6:**
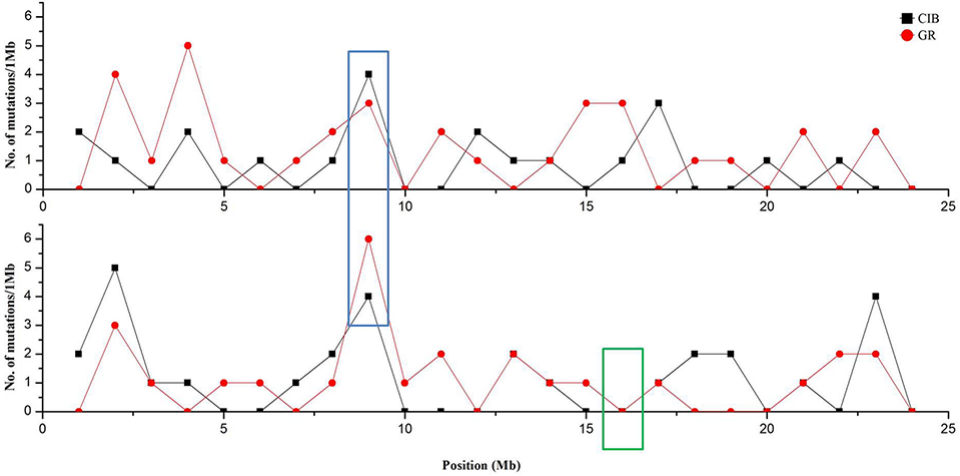
Clustered mutation regions on Chr. 9 and Chr.10 (in blue box) and no mutation region on Chr. 10 (in green box). Red line and dots represent GR-induced mutations, and black line and squares represent CIB-induced mutations.

Although these mutations were almost evenly distributed across the genome, unusual high-frequency (HF) mutation regions were discovered by analyzing the mutation frequency per Mb along the genome. For example, the GR-induced mutations in the region from 16 to 17 Mb accounted for 15.22% of all mutations on Chr. 4, and the mutation frequency within this region was 7 × 10^−6^ which was much higher than the average mutation frequency 1.2 × 10^−6^ on Chr. 4. The same anomaly was also observed in the region from 1 to 2 Mb for CIB-induced mutations on Chr. 10 with the mutation frequency 5 × 10^−6^ which was higher than the average mutation frequency 7.6 × 10^−7^ on Chr. 10 (**Figure 5**). Some common HF mutation regions occurred simultaneously for both the CIB and GR treatments, e.g. 8 to 9 Mb on Chr. 9 and Chr. 10 (**Figure 6**). We also found low-frequency (LF) mutation regions along the genome. For example, no mutation was detected in the region from 16 to 17 Mb on Chr.10 for either treatment. The mutation frequency within the HF regions was significantly higher than the LF regions (*P* < 0.05).

### Theoretical Mutation Frequency of the M_1_ Generation

**Figure 7:**
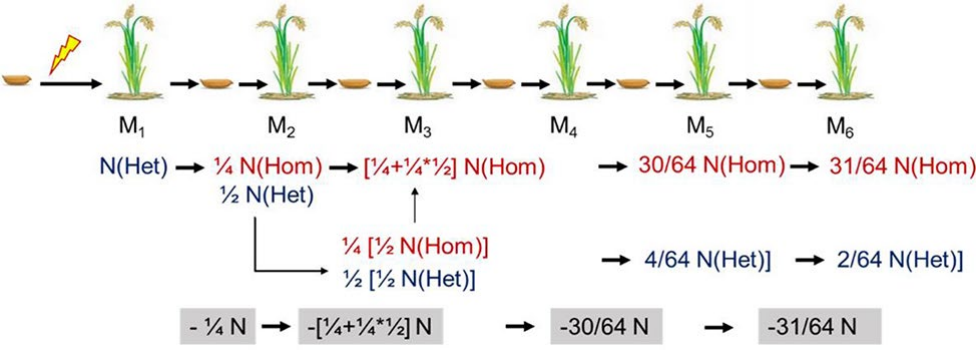
Theoretical genetic model of mutations in self-pollinated offspring. Het, heterozygous; Hom, homozygous.

The mutants for resequencing in our study were in M_4_, M_5_, or M_6_. For a self-pollinated plant, only 25% of the mutations can be permanently preserved in the offspring. Therefore, we could roughly estimate the mutation rate in M_1_ according to the probability of heritable mutations in different generations (**Figure 7**). To facilitate this calculation, we assumed that the number of lost mutations was equal to the number of homozygous mutations. Then the probable mutation rate in M_1_ was calculated by the following formula: No. homologous mutations × 2 + no. heterozygous mutations. Thus, we inferred that the mutation rate induced by CIBs was 2.4 × 10^−7^, and the mutation rate induced by GRs was 5.0 × 10^−7^ (**Table 3**) similar to previously reported the mutation rate (Du et al., 2017).

**Table 3:** Estimated numbers of mutations and mutation rate in M1 plants.

Treatments	Mutant	Generation for resequencing	No. of mutations	Homozygous mutations	Heterozygous mutations	The estimated mutations in M1	Average number and mutation rate in M^1^
CIB	H633	M4	52	44	8	96	89.67, 2.4 × 10−7
	H634	M4	46	44	2	90	
	H635	M5	51	45	6	96	
	H636	M5	71	59	11	129	
	H639	M4	35	34	1	71	
	H640	M4	28	28	0	56	
GR	H404	M6	109	93	16	202	185.25, 5.0 × 10−7
	H409	M6	109	89	20	198	
	H410	M6	94	84	10	178	
	H411	M6	69	74	15	163	

Mutation rate was calculated based on the Japonica genome size (373,245,519 bp).

### Characteristics of Mutations Induced by CIB and GR Irradiation

#### SBSs Induced by CIB and GR Irradiation

**Figure 8:**
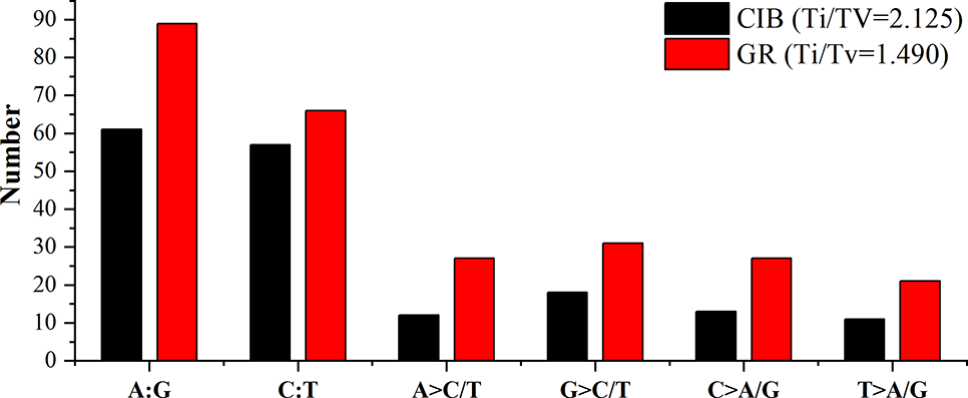
Transitions and transversions of induced by CIB irradiation and GRs. A:G and C:T are transitions. A > C/T, G > C/T, C > A/G, and T > A/G are transversions.

Exposure to CIB and GR irradiation induced many substitutions. SBSs were the most abundant variants induced by both CIB irradiation and GRs. Two categories transitions (mutations that occur among the same types of bases, i.e. purine > purine or pyrimidine > pyrimidine) and transversions (mutations that occur among the different types of bases, i.e. purine > pyrimidine or pyrimidine > purine) were identified for both mutagens. More transitions than transversions were induced by both mutagens (**Figure 8**). A total of 175 CIB-induced substitutions were identified and classified, yielding a transition to transversion (Ti/Tv) ratio of 2.22, and the Ti/Tv ratio of GR-induced substitutions was 1.49.

Two kinds of transitions, the G > A transition and C > T transition, were frequently induced by both mutagens. Fifty-five of the 259 detected GR-induced SBS mutations were G > A transitions, and 42 were C > T mutations; and 34 of the 175 detected CIB-induced SBS mutations were G > A transitions, and 37 were C > T mutations. The flanking DNA sequences of the most prominent substitutions of G > A transitions were analyzed, and 28 (82.35%) of these occurred at purine dinucleotide sites for CIB-induced mutations (**Table S2**), and 38 (69.09%) of these occurred at purine dinucleotide sites for GR-induced mutations (**Table S3**). For the substitutions of the C > T transitions, 27 (72.97%) of these occurred at pyrimidine dinucleotide sites for CIB-induced mutations (**Table S4**), and 27 (64.29%) of these occurred at pyrimidine dinucleotide sites for GR-induced mutations (**Table S5**).

At the genome-wide scale, 68.00% of the 175 CIB-induced SBSs occurred in upstream and downstream regions, 1.14% occurred in 3′/5′-untranslated regions (UTRs), 20.00% occurred in exon, 6.29% occurred in intron, and 4.57% occurred in the intergenic regions (**Table S6**). A total of 54.34% of the 259 GR-induced SBSs occurred in upstream and downstream regions, 3.65% in 3′/5′-UTRs, 25.00% occurred in exon, 1.83% occurred in splice regions, 8.68% occurred in intron, and 6.39% occurred in intergenic regions (**Table S6**). Notably, most of the SBSs occurred within the upstream or downstream region of genes, especially within upstream regions for CIB (51.43%) and GR (54.34%) irradiation. The upstream region of genes contains the active transcriptional region, and its sequence features are conserved to some extent. It was inferred that these regions may be more susceptible to free radicals induced by radiation.

### InDels Induced by CIB and GR Irradiation

We detected 72 InDels induced by CIB irradiation, and the vast majorities (83.10%) were deletion mutations. A total of 68 InDels were induced by GRs, 77.94% of which were deletion mutations. Notably, multiple base deletions (≥5 bp) were induced at a much higher frequency by CIBs than by GRs (**Figure S1**).

A total of 68.06% of the 72 CIB-induced InDels occurred in upstream and downstream regions, 5.56% occurred in 3′/5′-UTRs, 13.89% occurred in exons, 5.56% occurred in introns, and 2.78% occurred in intergenic regions (**Table S7**). A total of 72.06% of the 67 GR-induced InDels occurred in upstream and downstream regions, 2.94% occurred in the 3′/5′-UTRs, 13.24% in exons, 10.29% occurred in introns, and 1.47% occurred in intergenic regions (**Table S7**).

Similar to SBSs, most of InDels induced by the two treatments occurred within upstream or downstream region of genes, especially within upstream regions for CIB (55.56%) and GR (47.05%) irradiation. For the InDels within upstream regions, 82.5% of InDels were induced by CIBs, and 84.4% of SBSs induced by GRs occurred upstream of functional genes.

### MNVs Induced by CIB and GR Irradiation

**Figure 9:**
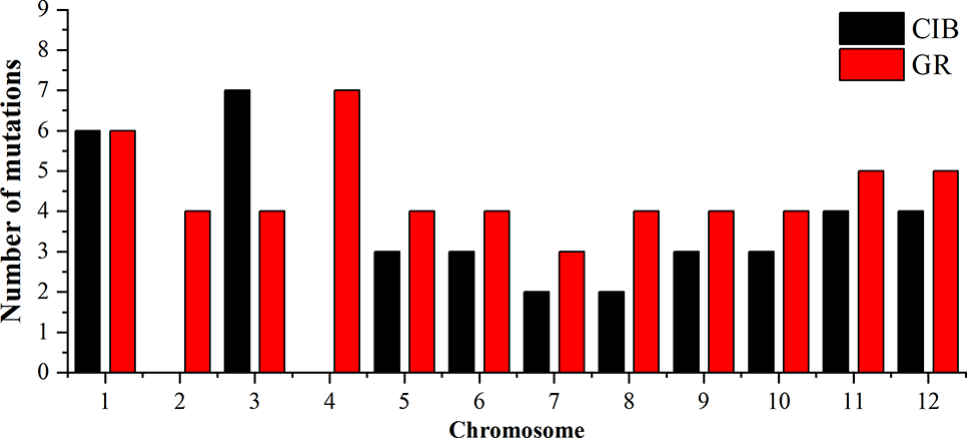
MNVs on different chromosome induced by CIB irradiation and GRs.

**Figure 10:**
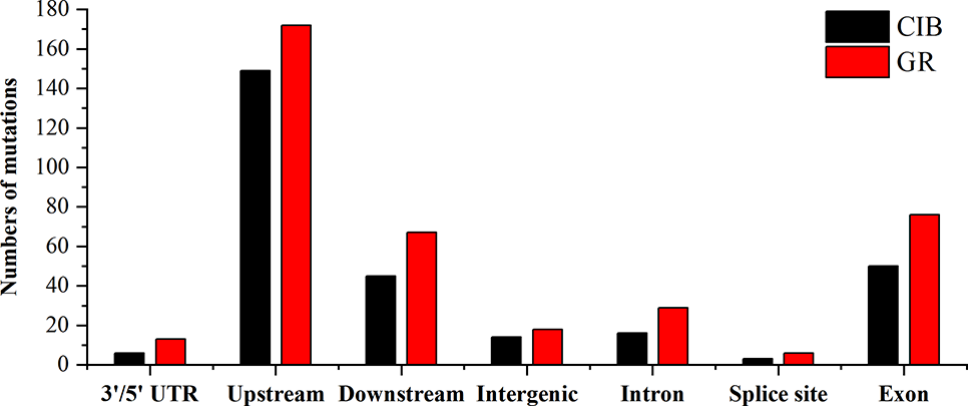
The locations on the genome of mutations induced by CIB irradiation and GRs.

MNVs are clustered mutations. We found 36 MNVs induced by CIBs and 54 MNVs induced by GRs. No MNVs were observed in Chr. 2 and Chr. 4 induced by CIBs (**Figure 9**). The MNVs occurred at a relatively high frequency within upstream and downstream regions for both mutagens (**Figure 10**). The MNVs in upstream regions accounted for 52.78% and 38.89% of MNVs induced by CIBs and GR, respectively. In addition, 13.89% of the 37 CIB-induced MNVs occurred in exons, 2.78% occurred in introns, and 11.11% occurred in intergenic regions (**Table S8**). A total of 5.56% of the 54 GR-induced MNVs were located in 3′/5′-UTRs, 22.22% were located in exon, 5.56% were located in introns, and 5.56% were located in intergenic regions (**Table S8**).

### Effects of Radiation-Induced Variation on Gene Function

All of the detected mutations were analyzed *via* SnpEff, which is referred to as the Japonica transcriptome database to predict the possible effects of mutations on gene function. Three types of mutations were recognized based on their effect on the genome including missense, nonsense, and silent mutations. The predicted effects of all mutations on gene function are shown in **Table 4**. 57.83% and 2.67% of the mutations induced by GRs led to missense and nonsense, respectively, and 58.14% of the mutations induced by CIBs led to missense. The silent mutations accounted for a higher proportion under CIB treatment (41.86%) than that under GR treatment (24.19%). Taken together, the results showed that more than half of the mutations had affected gene functions, and only 1.27% on average may have caused phenotypic changes.

**Table 4:** The predicted effects of mutations on gene function.

Category	Classification	GR		CIB	
		No.	Percentage	No.	Percentage
Effects of mutation on gene function	Missense	43	69.00%	25	58.14%
	Nonsense	4	6.45%	0	0.00%
	Silent	15	24.19%	18	41.86%

Further analyses revealed that the functions of 4.75 and 1.1 genes on average in each mutant were predicted by SnpEff to be highly affected by the mutations induced by CIBs and GRs, respectively (**Table 5**). These mutations caused stop gain or stop lost in gene translation and would affect essential function of proteins. Seven genes in both H410 and H411 treated with GR were predicted as being greatly affected in gene functions with stop gain or stop lost. Rare functionally affected genes were founded in the mutants treated with CIBs. Three mutants, H636, H693, and H640, even presented no such genes, whereas they all had phenotype of reduced seed set. In general, the number of mutations speculated to affect gene function accounted for 4.2% of all mutations, i.e. for every 100 mutations, approximately four stop-gain or stop-lost mutations were likely to highly affect gene functions.

**Table 5:** Functionally affected genes in each mutant.

Treatment	Line	Phenotype description	No. of functionally affected genes	Gene ID
CIBs	H633	Lower seed set	2	LOC_Os03g54860, LOC_Os07g02590
	H634	Lower seed set	4	LOC_Os01g03260, LOC_Os01g20170, LOC_Os08g27590, LOC_Os08g27590
	H635	Lower seed set	1	LOC_Os02g05850
	H636	Lower seed set	0	0
	H639	Lower seed set	0	0
	H640	Lower seed set	0	0
GRs	H404	Abnormal floret	4	LOC_Os01g33380, LOC_Os07g34180, LOC_Os09g24480, LOC_Os09g25660
	H409	Semi-dwarf	1	LOC_Os06g25900
	H410	Semi-dwarf	7	LOC_Os06g25900, LOC_Os03g20180, LOC_Os03g32610, LOC_Os03g53400, LOC_Os05g02580, LOC_Os06g06310, LOC_Os10g18510, LOC_Os10g28310
	H411	Semi-dwarf	7	LOC_Os01g39210, LOC_Os02g25500, LOC_Os07g09450, LOC_Os07g29730, LOC_Os07g39300, LOC_Os09g18110, LOC_Os10g15279

### Mapping of Causal Mutation in the Mutant Line H404

The GR-induced mutant H404 displayed abnormal flower development. H404 was crossed with 02428 (with normal floret), and the F_2_ population was constructed. Eleven pairs of SSR markers were found to be polymorphic between H403 and 02428 and were used for following BSA. The ratio of plants with normal spikelets and abnormal spikelets from the F_2_ (H404/02428) was in accordance with the theoretical proportion 3:1 (458:138, χ^2^ = 1.08), which indicated that abnormal flower development was controlled by a single recessive gene. BSA of the D pool and the R pool using the above 11 pairs of polymorphic SSR markers revealed that, only one marker RM566 was found to be polymorphic between the two pools. This phenotype-associated marker was near the physical location of 14704798 on Chr. 9. Then we investigated the nonsense mutations around this marker in the resequencing data and an 8-bp deletion locating upstream of RM566 were screened out. Our resequencing data showed that the 8-bp deletion occurred in the first exon of LOC_Os09g24480, resulting in frameshift mutation and premature termination of translation according to the SnpEff prediction. LOC_Os09g24480 encodes a TCP family transcription factor defining the diversification of floral morphology in rice and is highly expressed in palea primordium during early flower development (Yuan et al., 2009). Further analyses revealed that LOC_Os09g24480 is the previously reported cloned gene *RETARDED PALEA1* controlling palea development and floral zygomorphy in rice (Yuan et al., 2009; Zeng et al., 2016). Taken all together, we deduced that the 8 bp deletion in LOC_Os09g24480 was the causal mutation of the mutant line H404. So Mapping of the mutated gene through the combination of BSA and resequencing on the one hand proves the reliability of our resequencing data, and on the other hand provides another way of quick mapping of mutated gene.

## Discussion

In this article, we investigated and compared the difference mutation profiles induced by CIBs and GRs in rice using whole-genome resequencing technology. Generally, the genomic mutation profiles induced by CIBs and GRs were similar not only in mutation types but also in distribution on the chromosomes. Both CIB and GR irradiation induced SBSs, InDels, and MNVs. SBSs were the most abundant type of mutation, similar to studies of CIB- and GR-induced mutations in other plants, such as *Arabidopsis* and *Brachypodium* (Du et al., 2017; Lee et al., 2017). However, differences also existed in some aspects due to different physical characteristics of the two mutagens.

### Single Base Transitions Were the Main Form of SBSs

SBSs were the most abundant type of mutation induced by both mutagens, whereas more SBSs were induced by GRs than by CIBs, which was also observed in a study of mutational effects of GRs and CIBs on Arabidopsis seedlings (Yoshihara et al., 2013). Our research also shows that more transitions than transversions were detected in both CIB- and GR-induced mutations,which is consistent with previous reports. The Ti/Tv ratio of CIB-induced substitutions was 2.22, which is close to the 2.73 of spontaneous substitutions reported in the mutation accumulation line (Ossowski et al., 2010). Comparatively, the Ti/Tv ratio of GR-induced substitution was 1.49 indicating GRs may be inclined to induce more transversions. Radiation creates DNA lesions through the direct deposition of energy in the DNA as well as through the indirect action of reactive chemical species formed near the DNA attributed to the formation of hydroxyl radicals (·OH) through the radiolysis of water near (<10 nm) the DNA (Georgakilas et al., 2013). Elevated ROS are likely to induce 7,8-dihydro-8-oxoguanine (8-oxo-dG), which can form base pairs with adenine instead of cytosine during DNA replication and leads to GC TA transversions (Amoroso et al., 2011; Chen et al., 2012). The higher frequency of induced tranversions by GRs than by CIBs indicates in a way direct effect and indirect effect might play different roles under the two physical mutagens.

In the present study, C > T transitions mainly occurred at pyrimidine dinucleotide sites, which is consistent with the results of Du et al. (2017). Radiation exposure could induce C > T transitions by causing the formation of covalent linkages between neighboring pyrimidine residues (for instance, CC, CT, TC, and TT) in the DNA sequence, thus resulting in a tendency to form C > T mutations at pyrimidine sequences (Daya-Grosjean and Sarasin, 2005). Such a tendency was also found in UV and fast neutron irradiation (Belfield et al., 2012). Therefore, different types of physical radiation, such as UV, GR, CIB, and neutron radiation, may follow the same biological mechanism.

### CIB Irradiation Induced Increased InDels, Whereas GRs Induced Increased MNVs

LET (the energy transferred per unit length, keV µm^−1^) represents the energy deposition of ionizing radiations on their per unit track. The most important physical difference between CIB irradiation and GRs is the greater LET in the former. The LET of GRs is 0.2 keV µm^−1^ and the LET of a heavy-ion beam used in biological research ranges from 22.5 keV µm^−1^ to 4,000 keV µm^−1^ (Ryuto et al., 2008), and the LET of CIB in the present study is 80 keV µm^−1^, which is a relatively high value for biological research. Accelerated particles produced by heavy-ion beams densely deposit their energy within a localized region along the particle path in a strikingly different way than GRs, which sparsely deposit their energy in a large targeted volume (Kazama et al., 2011). Heavy-ion beam irradiation shows higher relative biological effectiveness (RBE) of lethality and cell inactivation compared to the low-LET radiation of GRs (Lett 1992; Tanaka et al., 2010). Therefore, significant DNA damage is likely to be caused by heavy-ion irradiation. It has been suggested that heavy-ion beams predominantly induce DSBs (Ward, 1994; Goodhead 1995) and a high yield of DSBs after heavy-ion beam irradiation was revealed by experiments on both animal and plant cells (Hoglund et al., 2000; Yokota et al., 2007). A high frequency of DSBs is likely to lead to severe DNA structure damage, such as InDels and cluster lesions.

In our study, CIB-induced InDels accounted for 25.44% of the total variations, while GR-induced InDels accounted for 17.85%. Du et al. (2017) and Lee et al. (2017) showed that heavy ion radiation induces a higher frequency of InDels than GRs, the probability of InDels induced by heavy-ion radiation is approximately 4.6 times that of GRs. Especially, we found large fragment deletion (≥5bp) occurred much frequently under CIB irradiation. We speculate that the concentration of ionization energy of CIB tends to induce large InDels. MNV, i.e. clustered mutation, was also induced genome wide by both CIBs and GRs in our study. However, inconsistent with the existing theory, we found that the frequency of MNVs was not necessarily related to the level of LET radiation. In fact, GRs with a low LET induced more MNVs than CIBs, which is typical high-LET irradiation; the frequency of MNVs induced by GRs was approximately 2.35 times that induced by CIBs. As we mentioned before, direct effect through the deposition of energy in the DNA as well as indirect effect through elevated ROS might play different roles in the cells under different types of radiation. For low-LET radiations, a large amount of mutations arise from indirect effects. The increased MNVs induce by low-LET GRs might be due to greater indirect effects.

### Mutations Induced by CIB Irradiation and GR Are Unevenly Distributed on Chromosomes

Chromosomes are long molecules that must be faithfully replicated and segregated during each cell cycle to provide genetic information to offspring (Murray and Szostak, 1985). For plant breeding, the most commonly used physical mutagenesis material is dry seed (Luo et al., 2016; Yamaguchi, 2018). The cells of dry seeds are in the interphase of division, and their chromosomes are in a complex three-dimensional reticular chromatin structure(Murray and Szostak, 1985). In radiation treatment, energy precipitates on the chromatin along with the radiation track, which leads to clusters of DNA lesions. Studies on plant mutants such as *A. thaliana* obtained by physical radiation did not indicate the characteristics of uneven distribution of mutations, which may be because these mutants reflect only a small amount of DNA variation retained by heredity and cannot fully reflect the initial damage caused by physical radiation. In addition, the depth of sequencing may also affect the analysis of DNA variation. It is helpful to understand the nature of physical radiation more comprehensively by deep sequencing multiple mutants and analyzing the characteristics of all mutation data.

The mutations were reliably sequenced, and the DNA mutations of six CIB-induced mutants and four GR-induced mutants were comprehensively analyzed in present study. The radiation-induced mutations were unevenly distributed across the chromosome. For example, the GR induced mutations in the region from 16 to 17 Mb accounted for 15.22% of all mutations on Chr. 4. The same anomaly was also observed in the region from 1 to 2 Mb for CIB-induced mutations on Chr. 10. Moreover, we found that 52.65% and 45.14% of the mutations induced by CIBs and GRs, respectively, were located in upstream regions of genes. It has been known for decades that the genomic loci, known as "common fragile sites" (CFS), are particularly prone to breakage and instability during mitosis (Arlt et al., 2006). Recently, owing to high-throughput genomic techniques, two classes of fragile sites were identified: (1) long, active genes and (2) promoters of transcriptionally active genes (Yang et al., 2015; Lensing et al., 2016). The clusters of mutation regions found in this study occurred more frequently the upstream regions of coding genes, which is in agreement with the characteristics of CFS. However, more mutation sequencing data are needed to prove whether there are mutation hotspots in chromosome molecular for physical radiation. We plan to use a single cell line of rice to study the initial DNA damage characteristics induced by physical radiation, and to track the generational transmission of these variations to further analyze the molecular characteristics of physical radiation.

## Data Availability Statement

All data generated or analyzed during this study are included in this published article/**Supplementary Material**.

## Author Contributions

TG and GY designed the project, WLu performed the sequencing and GY wrote the manuscript. JZ and XY assisted in mutants screening and data analysis. YD, LZ, and WLi conducted the CIB irradiation experiments and provided constructive suggestions. HW and ZC participated in the design of the work and reviewed the manuscript. All authors read and approved the final manuscript.

## Funding

Financial support for this research was provided in part by a grant from the National Key Technology Research and Development Program of China (No. 2016YFD0102102), the key Research and development Project of Guangdong Province (No. 2018B020206002) and the earmarked fund for Modern Agro-Industry Technology Research System (No. CARS-01-12).

## Conflict of Interest

The authors declare that the research was conducted in the absence of any commercial or financial relationships that could be construed as a potential conflict of interest.
