# Supplementary material for: Genome-Wide Comparisons of Mutations Induced by Carbon-Ion Beam and Gamma-Rays Irradiation in Rice via Resequencing Multiple Mutants
Source: Front Plant Sci. 2019 Nov 28;10:1514. doi: 10.3389/fpls.2019.01514 (PMC6892775; doi:10.3389/fpls.2019.01514)
Supplement: Supplementary file 1 [file DataSheet_1.docx]

Figure S1 InDels of various sizes induced by CIB and GR irradiation and gamma rays.

**Table S1 Variant rate across each chromosome induced by CIB and gamma rays**

|  | Chr. 1 | | Chr. 2 | | Chr. 3 | | Chr. 4 | | Chr. 5 | | Chr. 6 | |
| --- | --- | --- | --- | --- | --- | --- | --- | --- | --- | --- | --- | --- |
|  | Variants^a^ | Rate^b^ | Variants | Rate | Variants | Rate | Variants | Rate | Variants | Rate | Variants | Rate |
| CIB | 39 | 1109511 | 31 | 1159266 | 32 | 1137932 | 17 | 2088394 | 18 | 1664357 | 29 | 1077544 |
| GR | 45 | 961576.1 | 41 | 876518.3 | 44 | 827586.8 | 46 | 771797.7 | 20 | 1497922 | 40 | 781219.7 |
|  | Chr. **7** | | Chr. **8** | | Chr. **9** | | Chr. **10** | | Chr. **11** | | Chr. **12** | |
|  | Variants^a^ | Rate^b^ | Variants | Rate | Variants | Rate | Variants | Rate | Variants | Rate | Variants | Rate |
| CIB | 18 | 1649868 | 19 | 1497001 | 21 | 1095844 | 29 | 800251 | 15 | 1934740 | 15 | 1835457 |
| GR | 22 | 1349892 | 29 | 980793.9 | 33 | 697355.2 | 26 | 892588 | 20 | 1451055 | 12 | 2294321 |

A. Variants indicate the number of mutations in each chromosome. B. Rate equals to length of chromosome/variants.

Table S2 Pyrimidine dinucleotide analyses at G > A substitutions sites induced by CIB irradiation

| **Chromosome** | **Loci** | **Mutation** | **Flanking sequence** | **Pur-Pur** |
| --- | --- | --- | --- | --- |
| chr01 | 601170 | G>A | CTGTT**G**CTCCT |  |
| chr01 | 10944438 | G>A | CCGAA**G**AAATA | AA **G** AA |
| chr01 | 17480463 | G>A | ATCTT**G**GGACG | **G** GG |
| chr01 | 38661537 | G>A | GGGGC**G**GATGG | **G** GA |
| chr01 | 38888180 | G>A | TTTTG**G**TTACA | G **G** |
| chr01 | 39694340 | G>A | TTGAG**G**GTTAC | G **G** G |
| chr02 | 4810102 | G>A | AATTG**G**TCTTT | G **G** |
| chr02 | 9611857 | G>A | GAAAT**G**GTTGG | **G** G |
| chr02 | 14190414 | G>A | CCCCC**G**GGTAT | **G** GG |
| chr02 | 14788514 | G>A | CTTGC**G**GATAC | **G** GA |
| chr03 | 14763187 | G>A | TCTAG**G**ATTTT | G **G** A |
| chr04 | 15094272 | G>A | TGGTG**G**CGACG | G **G** |
| chr04 | 30150876 | G>A | ATGTG**G**GACAA | G **G** GA |
| chr05 | 10080074 | G>A | ACCGA**G**GATCA | A **G** GA |
| chr05 | 12153495 | G>A | TACTC**G**GAAAG | **G** GA |
| chr05 | 17105487 | G>A | CCAAG**G**AAATG | **G** AA |
| chr06 | 158848 | G>A | GCGTC**G**CCGTC |  |
| chr06 | 3125570 | G>A | ATCTC**G**CCATC |  |
| chr06 | 8819758 | G>A | AAGGG**G**TGACA | GG **G** |
| chr06 | 14066842 | G>A | TGACG**G**GCCCC | G **G** G |
| chr06 | 18189589 | G>A | GCATT**G**GGGAG | **G** GG |
| chr06 | 21749482 | G>A | ATTTA**G**ATCTA | A **G** A |
| chr06 | 29648780 | G>A | CAGCT**G**ACTAT | **G** A |
| chr07 | 589381 | G>A | TCCTC**G**GTGGC | **G** G |
| chr07 | 10592362 | G>A | GGCGC**G**CAGTG |  |
| chr07 | 21760542 | G>A | CCGTT**G**CTTCA |  |
| chr08 | 17304988 | G>A | CGTTC**G**GGAGC | **G** GG |
| chr09 | 5215200 | G>A | CAGTC**G**TACAA |  |
| chr09 | 15488340 | G>A | ACGGC**G**GACGG | **G** GA |
| chr10 | 1979422 | G>A | GAATT**G**GGCCA | **G** GG |
| chr10 | 12262037 | G>A | GTGGC**G**GAGGA | **G** GA |
| chr10 | 22412706 | G>A | AAGGT**G**AGAAG | **G** AG |
| chr11 | 6478983 | G>A | GCTCC**G**GGCCT | **G** GG |
| chr12 | 18412069 | G>A | TAATG**G**TATCA | G **G** |

Table S3 Pyrimidine dinucleotide analyses at G > A substitutions sites induced by gamma rays

| Chromosome | Loci | Mutation | Flanking sequence | Pur-Pur |
| --- | --- | --- | --- | --- |
| chr01 | 11643805 | G>A | CTGTC**G**AGAAC | **G**A |
| chr01 | 12383568 | G>A | GCCGA**G**ACGAC | A**G**A |
| chr01 | 13299553 | G>A | GTAAA**G**GGTGG | A**G**G |
| chr01 | 17201671 | G>A | GCACA**G**TAGTG | A**G** |
| chr01 | 21276028 | G>A | TAAGA**G**TGATA | A**G** |
| chr01 | 22317151 | G>A | ACGGC**G**GTCAC | **G**G |
| chr01 | 33813817 | G>A | AGATT**G**GACCA | **G**G |
| chr01 | 41835668 | G>A | TCTAG**G**CTCTA | G**G** |
| chr02 | 8698651 | G>A | AGCTT**G**GCAAG | **G**G |
| chr02 | 13492726 | G>A | TATCT**G**GAGTA | **G**G |
| chr02 | 25522227 | G>A | AAGCG**G**CAGAA | G**G** |
| chr02 | 29380674 | G>A | ATCGA**G**CGAAT | A**G** |
| chr02 | 31987531 | G>A | TGATT**G**TGTTA |  |
| chr03 | 5232037 | G>A | GTTAG**G**ACTGC | G**G**A |
| chr03 | 5257844 | G>A | TCACT**G**ACTCT | **G**A |
| chr03 | 13044300 | G>A | ATGGC**G**TTTTC |  |
| chr03 | 16490782 | G>A | AAACC**G**TTTTG |  |
| chr03 | 16802414 | G>A | AGATC**G**TAAGA |  |
| chr03 | 16931654 | G>A | TTCCC**G**GTTGG | **G**G |
| chr03 | 18663646 | G>A | CGTCT**G**CCGCC |  |
| chr03 | 19643255 | G>A | CGATT**G**TCGGC |  |
| chr03 | 25763465 | G>A | CCTCG**G**TATCT | G**G** |
| chr03 | 26170893 | G>A | TCGGG**G**GATTT | G**G**G |
| chr04 | 5937855 | G>A | TGAAT**G**TTAAA |  |
| chr04 | 11865868 | G>A | CTATC**G**TCTAC |  |
| chr04 | 16195105 | G>A | ACCGG**G**ACTAA | G**G**A |
| chr04 | 16874783 | G>A | AACTG**G**GACTA | G**G**G |
| chr04 | 26211775 | G>A | GCCTT**G**GAATT | **G**G |
| chr04 | 33480253 | G>A | TCGCA**G**AGGAG | A**G**A |
| chr05 | 7604673 | G>A | TGGAG**G**TCTTG | G**G** |
| chr05 | 22545776 | G>A | ACTAC**G**CGTGG |  |
| chr05 | 22706405 | G>A | TCAAT**G**CTACA |  |
| chr05 | 27752250 | G>A | CTTCA**G**AGCTA | A**G**A |
| chr06 | 1167436 | G>A | CGAGC**G**CAAGG |  |
| chr06 | 12980473 | G>A | AACAT**G**TTTAA |  |
| chr06 | 13318140 | G>A | CTACT**G**ACGTC | **G**A |
| chr06 | 13405129 | G>A | TCAGG**G**TTACG |  |
| chr06 | 21596188 | G>A | AAGTC**G**GATAA |  |
| chr06 | 21861283 | G>A | GTCAT**G**TTGAA |  |
| chr06 | 22419962 | G>A | TAAAA**G**CCCGT | A**G** |
| chr06 | 22419991 | G>A | GTGAC**G**GGCAT | **G**G |
| chr07 | 5355954 | G>A | CATCA**G**ATTTT | A**G**A |
| chr07 | 12114919 | G>A | AGTTC**G**CCGTG |  |
| chr08 | 12068534 | G>A | AAACA**G**GGACT | A**G**G |
| chr08 | 13561456 | G>A | TGGGC**G**GAGAT | **G**G |
| chr08 | 24037712 | G>A | TCATG**G**GACAA | G**G**G |
| chr09 | 3208547 | G>A | GTCGG**G**CGCAA | G**G** |
| chr09 | 3733307 | G>A | CGCCG**G**AGGAC | G**G**A |
| chr09 | 7748156 | G>A | GGAGC**G**AATTT | **G**A |
| chr09 | 8089774 | G>A | GGAAC**G**AAGCA | **G**A |
| chr10 | 1363936 | G>A | GCAGG**G**GGTTT | G**G**G |
| chr10 | 8896119 | G>A | AGGCG**G**CAAGG | G**G** |
| chr10 | 8896124 | G>A | GCAAG**G**CGGTC | G**G** |
| chr10 | 22053759 | G>A | GCTAT**G**CTTAA |  |
| chr11 | 13271297 | G>A | GAAGA**G**CATAA | A**G** |

Table S4 Pyrimidine dinucleotide analyses at C > T substitutions sites induced by CIB irradiaiton

| Chromosome | Loci | Mutation | Flanking sequence | Pur-Pur |
| --- | --- | --- | --- | --- |
| chr01 | 16147255 | C>T | CCGTC**C**GGAAA |  |
| chr01 | 33941886 | C>T | TTGAG**C**GCCGA |  |
| chr01 | 38740293 | C>T | ACGGC**C**ATACC |  |
| chr02 | 11281774 | C>T | TCAAT**C**GGCTC | T**C** |
| chr03 | 16049895 | C>T | CTGAT**C**GGCAA | T**C** |
| chr03 | 17568612 | C>T | ATAAG**C**TGTTC | **C**T |
| chr03 | 26254680 | C>T | GAGAG**C**AAGAG |  |
| chr03 | 27394070 | C>T | GGGTG**C**GACGG |  |
| chr04 | 10862808 | C>T | GTCGC**C**GCCAA | C**C** |
| chr04 | 17410286 | C>T | ATGGT**C**GGAAG | T**C** |
| chr04 | 20735726 | C>T | CTTTT**C**TGCAG | T**C**T |
| chr04 | 24745782 | C>T | ATGGA**C**GATCA |  |
| chr04 | 29496431 | C>T | AATCA**C**GGTTT |  |
| chr04 | 32077793 | C>T | TCGTA**C**AAAAA |  |
| chr05 | 13675467 | C>T | AAAAC**C**CAATT | C**C**C |
| chr05 | 23458948 | C>T | TCTCT**C**TAGGC | T**C**T |
| chr06 | 10757154 | C>T | CGTCG**C**TCGCC | **C**TC |
| chr06 | 19447904 | C>T | TTTCA**C**CCTTA | **C**CC |
| chr06 | 26537495 | C>T | CCTTA**C**TCTTG | **C**TC |
| chr07 | 1893473 | C>T | CAGGC**C**TCCGG | C**C**T |
| chr07 | 10538317 | C>T | TGCTG**C**GATTA |  |
| chr07 | 19437750 | C>T | CCCTC**C**GCGCT | TC**C** |
| chr07 | 24597153 | C>T | GCCTA**C**CAAAT | **C**C |
| chr07 | 24597154 | C>T | CCTAC**C**AAATG | C**C** |
| chr08 | 15511100 | C>T | CATGG**C**CAGCT | **C**C |
| chr08 | 24414444 | C>T | TGCAC**C**GGTGA | C**C** |
| chr09 | 11823387 | C>T | ATCTG**C**CAGGA | **C**C |
| chr09 | 12860653 | C>T | TTGAT**C**GGTGG | T**C** |
| chr10 | 8228935 | C>T | GTCCC**C**ATTTA | C**C** |
| chr10 | 17011197 | C>T | TTTGA**C**TCTCT | **C**T |
| chr10 | 17839429 | C>T | AACAT**C**AACCT | T**C** |
| chr10 | 18162781 | C>T | TTCGG**C**TGTGT | **C**T |
| chr10 | 20382494 | C>T | CCTTG**C**GGGTT |  |
| chr11 | 984153 | C>T | AGTTC**C**GGACC | C**C** |
| chr11 | 6498639 | C>T | CTGTC**C**ATCAG | C**C** |
| chr11 | 11473157 | C>T | GCTGC**C**GACTT | C**C** |
| chr12 | 462131 | C>T | GGAAT**C**CAAGC | T**C**C |

Table S5 Pyrimidine dinucleotide analyses at C > T substitutions sites induced by gamma rays

| Chromosome | Loci | Mutation | Flanking sequence | Pur-Pur |
| --- | --- | --- | --- | --- |
| chr01 | 18164211 | C>T | CGCGG**C**GTAGT |  |
| chr01 | 37868997 | C>T | ACGGG**C**AACGG |  |
| chr02 | 22942232 | C>T | ATACC**C**ATAAC |  |
| chr02 | 29157098 | C>T | GTTGT**C**TGCTA | T**C**T |
| chr03 | 12359183 | C>T | TAAAA**C**TTTTG | **C**T |
| chr03 | 13048921 | C>T | GATTT**C**CTTGT | T**C**C |
| chr03 | 14609324 | C>T | TCTGA**C**AGTGA |  |
| chr03 | 22235095 | C>T | TTTAT**C**AGATT | T**C** |
| chr03 | 24471077 | C>T | ATGCA**C**TGTGT | **C**T |
| chr04 | 11512293 | C>T | TCTAT**C**AAATG |  |
| chr04 | 22346535 | C>T | CAGTG**C**GGAGT |  |
| chr05 | 9359837 | C>T | CCCTC**C**GCAAG |  |
| chr05 | 12293639 | C>T | ACTGC**C**TTTAT | **C**T |
| chr05 | 13693473 | C>T | TTAAA**C**AACTT |  |
| chr05 | 15067570 | C>T | CGACA**C**GTAGC |  |
| chr06 | 429959 | C>T | AATCA**C**ATATC |  |
| chr06 | 12723246 | C>T | GGTAT**C**CCCTC | T**C**C |
| chr06 | 17601157 | C>T | ACTCC**C**ATAGT | C**C** |
| chr06 | 22419984 | C>T | GTCAT**C**TGTGA | T**C**T |
| chr07 | 2289186 | C>T | GAGCG**C**GCTCC |  |
| chr07 | 22840866 | C>T | AATGC**C**CCGGG | C**C**C |
| chr08 | 6014274 | C>T | TTCCC**C**GTGAA | C**C** |
| chr08 | 6062464 | C>T | TTTTT**C**ACCTG | T**C** |
| chr08 | 8524252 | C>T | CCATA**C**GACGA |  |
| chr08 | 15763490 | C>T | GCTCA**C**GAACC |  |
| chr08 | 24876807 | C>T | ACTCT**C**CCTCC | T**C**C |
| chr08 | 25679208 | C>T | AGCTC**C**ACAGC | C**C** |
| chr09 | 1479901 | C>T | ACAAG**C**CGATT | **C**C |
| chr09 | 2228852 | C>T | GACGA**C**GATGA |  |
| chr09 | 8056845 | C>T | TCCTC**C**GCATC | C**C** |
| chr09 | 13450078 | C>T | ACAAC**C**GAGGA | C**C** |
| chr09 | 20675847 | C>T | CAAAC**C**AGAAG | C**C** |
| chr10 | 8228382 | C>T | GATTT**C**TCTTA | TT**C**TC |
| chr10 | 10009696 | C>T | AAGTT**C**GGCTC | T**C** |
| chr10 | 13785262 | C>T | ATTTG**C**TCTCA | **C**TC |
| chr11 | 14563032 | C>T | TACAG**C**AGCGT |  |
| chr11 | 26108233 | C>T | ATTGT**C**TAGAT | T**C**T |
| chr12 | 1985002 | C>T | AATAT**C**TGCAA | T**C**T |
| chr12 | 3405414 | C>T | TAATT**C**CAACT | T**C**C |
| chr12 | 11302143 | C>T | ATATT**C**GAGGT | TT**C** |
| chr12 | 12232604 | C>T | AGGAT**C**TTCGA | T**C**TT |
| chr12 | 19865487 | C>T | CCCAC**C**GAGTC | C**C** |

**Table S6** Annotation of SBSs induced by CIB irradiation and gamma rays

| Treatments | 3'/5'UTR | Upstream/downstream | | Intergenic | Intron | Splice site | Exon |
| --- | --- | --- | --- | --- | --- | --- | --- |
|  |  | Upstream | Downstream |  |  |  |  |
| CIB | 2 | 90 | 29 | 8 | 11 | 0 | 35 |
| GR | 8 | 119 | 0 | 14 | 19 | 4 | 55 |

| **Table S7 Annotation of InDels induced by** CIB irradiation and gamma rays | | | | | | | |
| --- | --- | --- | --- | --- | --- | --- | --- |
| Treatments | 3'/5'UTR | Upstream/downstream | | Intergenic | Intron | Splice site | Exon |
|  |  | Upstream | Downstream |  |  |  |  |
| CIB | **4** | 40 | 9 | **2** | **4** | **3** | **10** |
| GR | **2** | 32 | 17 | **1** | **7** | **0** | **9** |

**Table S8** Annotation of MNVs induced by CIB irradiation and gamma rays

| Treatments | 3'/5'UTR | Upstream/downstream | | Intergenic | Intron | Splice site | Exon |
| --- | --- | --- | --- | --- | --- | --- | --- |
|  |  | Upstream | Downstream |  |  |  |  |
| CIB | 4 | 19 | 7 | 4 | 1 | 0 | 5 |
| GR | 3 | 21 | 10 | 3 | 3 | 2 | 9 |
